# Supplementary material for: Prevalence, Genetics and Evolutionary Properties of Eurasian Avian-like H1N1 Swine Influenza Viruses in Liaoning
Source: Viruses. 2022 Mar 20;14(3):643. doi: 10.3390/v14030643 (PMC8953428; doi:10.3390/v14030643)
Supplement: Supplementary file 1 [file viruses-14-00643-s001.zip › viruses-1598072-Supplementary Materials Revised.pdf]

viruses

MDPI

Supplementary Materials

Prevalence, genetics and evolutionary properties of Eurasian avian-like H1N1 swine influenza viruses in Liaoning

Hailing Li, Haoyu Leng, Siqi Tang, Chaofan Su, Yina Xu, Yongtao Wang, Jiaming Lv, Shiwei Zhang, Yali Feng, Shaokang Song and Ying Zhang *

Table S1. Pig nasal swabs sampling details in this study.

City	Sampling Month	Pig Farms and Its Production Modes	The Quantity of Live Hogs	Sampling Times	Positive /Total	Name of Representative Strain
JZ	Jan.	No.1: Wean to finish	4400	2	14/575，17/353	JZ164, JZ266
	Mar.	No.2: Wean to finish	6600	2	0/436，4/324	H1N2
	Jul.	No.1: Wean to finish	4400	1	5/600	H1N2
	Sept.	No.2: Wean to finish	6600	1	7/418	H1N2
	Oct.	No.3: Self-propagation pig farm with 600 sows	3000	1	8/457	H1N2
FS	Feb.	No.4: Wean to finish	12000	1	0/388	—
	May.	No.4/No.5: Wean to finish	12000/2400	2	19/726，8/407	FS1553, FS1084
	Nov.	No.4: Wean to finish	12000	1	0/613	—
PJ	Apr.	No.6: Self-propagation pig farm with 1000 sows	5000	1	0/335	—
	Mar.	No.7: Wean to finish	9600	2	15/518，18/905	PJ521, PJ626
	Aug.	No.7: Wean to finish	9600	1	0/247	—
	Nov.	No.8: Self-propagation pig farm with 1000 sows	5000	1	0/326	—
DL	Jan.	No.9: Wean to finish	12000	1	65/1510	DL1022
	Apr.	No.9: Wean to finish	12000	1	23/526	DL1007
	Jun.	No.9: Wean to finish	12000	1	0/551	—
	Sept.	No.9: Wean to finish	12000	1	0/668	—
	Dec.	No.9: Wean to finish	12000	1	4/726	DL5436
FX	Jan.	No.10: Wean to finish	5000	1	0/590	—
	Mar.	No.10: Wean to finish	5000	1	0/533	—
	Jun.	No.12: Self-propagation pig farm with 800 sows	3900	3	52/458，36/428，0/444	FX1635, FX1638
	Jul.	No.10: Wean to finish	5000	1	0/626	—
	Oct.	No.11: Self-propagation pig farm with 800 sows	3900	2	0/337，0/456	—
LY	Feb.	No.13: Self-propagation pig farm with 600 sows	3000	1	0/726	—
	Apr.	No.14: Wean to finish	4800	1	0/572	—
	Aug.	No.14: Wean to finish	4800	2	21/1437	LY3468
	Nov.	No.13: Self-propagation pig farm with 600 sows	3000	1	28/642	LY3478
AS	Jan.	No.15: Wean to finish	7200	1	0/286	—
	Feb.	No.15: Wean to finish	7200	1	0/314	—
	Apr.	No.15: Wean to finish	7200	1	0/352	—
	Jul.	No.15: Wean to finish	7200	1	23/833	AS1732
	Aug.	No.15: Wean to finish	7200	1	52/1216	AS1734
	Dec.	No.15: Wean to finish	7200	1	0/212	—
TL	Jan.	No.16: Wean to finish	6000	1	0/210	—
	Mar.	No.17: Self-propagation pig farm with 800 sows	3900	1	0/227	—
	Apr.	No.16: Wean to finish	6000	1	0/328	—
	Jul.	No.17: Self-propagation pig farm with 800 sows	3900	1	0/449	—
	Nov.	No.16: Wean to finish	6000	1	48/733	TL5239
	Dec.	No.17: Self-propagation pig farm with 800 sows	3900	1	6/626	TL5404

Table S1. Cont.

City	Sampling Month	Pig Farms and Its Production Modes	The Quantity of Live Hogs	Sampling Times	Positive /Total	Name of Representative Strain
CY	Mar.	No.18: Wean to finish	5000	1	0/500	—
	May.	No.19: Wean to finish	3000	1	0/588	—
	Sept.	No.18: Wean to finish	5000	1	13/833	CY1833
	Oct.	No.19: Wean to finish	3000	1	53/898	CY3424
HLD	Mar.	No.20: Self-propagation pig farm with 700 sows	3400	1	0/473	—
	Jul.	No.21: Wean to finish	4000	1	35/652	HLD1706
	Sept.	No.20: Self-propagation pig farm with 700 sows	3400	1	5/426	HLD1795
	Nov.	No.22: Wean to finish	4800	1	0/725	—
SY	Jan.	No.23: Wean to finish	2400	1	8/125	H1N2
	Feb.	No.24: Wean to finish	3000	1	1/269	SY369
	Mar.	No.25: Wean to finish	4800	1	6/454	H1N2
	Apr.	No.26:Self-propagation pig farm with 600 sows	3000	1	29/413	H3N2
	May.	No.27: Wean to finish	2400	1	3/266	H1N2
	Jun.	No.25: Wean to finish	4800	1	2/397	H1N2
	Jul.	No.28: Wean to finish	5000	1	0/179	—
	Aug.	No.24: Wean to finish	3000	1	12/478	SY1772
	Sept.	No.26:Self-propagation pig farm with 600 sows	3000	1	7/89	SY514
	Oct.	No.29: Wean to finish	4000	1	6/172	H1N2
	Nov.	No.25: Wean to finish	4800	1	5/322	H1N2
	Dec.	No.30: Wean to finish	3000	1	11/292	H1N2

Table S2. Primers were used in this study.

Primer Name	Primer Sequence (5′-3′)	Direction
Uni12	AGCAAAAGCAGG	Forward
AIV-M-F	AAGCGTCTACGCTGCAGTCC	Forward
AIV-M-R	TTCTAACCGAGGTCGAAAC	Reverse
PB2-amplification-F	AGCAAAAGCAGGTCAATTATATTC	Forward
PB2-amplification-R	AGTAGAAACAAGGTCGTTTTTAAACA	Reverse
PB1-amplification-F	AGCAAAAGCAGGTCAATTATATTC	Forward
PB1-amplification-R	AGTAGAAACAAGGCATTTTTTCAT	Reverse
PA-amplification-F	AGCAAAAGCAGGTACTGATCC	Forward
PA-amplification-R	AGTAGAAACAAGGTACTTTTTTGGA	Reverse
HA-amplification-F	AGCAAAAGCAGGGGATAATA	Forward
HA-amplification-R	AGTAGAAACAAGGGTGTTTTTTC	Reverse
NP-amplification-F	AGCAAAAGCAGGGTAGATAATC	Forward
NP-amplification-R	AGTAGAAACAAGGGTATTTTTATTC	Reverse
NA-amplification-F	AGCAAAAGCAGGAGTTCAAA	Forward
NA-amplification-R	AGTAGAAACAAGGAGTTTTTTGAAC	Reverse
M-amplification-F	AGCAAAAGCAGGTAGATATTTAAAG	Forward
M-amplification-R	AGTAGAAACAAGGTAGTTTTTTACTCC	Reverse
NS-amplification-F	AGCAAAAGCAGGGTGAC	Forward
NS-amplification-R	AGTAGAAACAAGGGTGTTTTTTATC	Reverse

Table S3. Serum sampling details in this study.

City	Sampling Month	Pig Farms and Its Production Modes	The Quantity of Live Hogs	Sampling Times	Positive /Total
JZ	Jan.	No.1: Self-propagation pig farm with 800 sows	5000	1	84/244
FS	Feb.	No.2: Wean to finish	12000	1	50/116
PJ	Apr.	No.3: Self-propagation pig farm with 600 sows	3000	1	15/30
DL	Jan.	No.4: Self-propagation pig farm with 600 sows	3000	1	19/81
FX	Jan.	No.4: Self-propagation pig farm with 600 sows	2000	1	11/23
LY	Feb.	No.6: Pig breeding farm	1000	1	20/87
AS	Jan.	No.7: Pig breeding farm	1200	1	99/100
TL	Jan.	No.8: Pig breeding farm	1200	1	91/316
CY	Mar.	No.9: Wean to finish	1500	1	29/90
HLD	Mar.	No.10: Wean to finish	1500	1	1/30
SY	Jan.	No.11: Self-propagation pig farm with 300 sows	1400	1	5/68
	Mar.	No.12: Pig breeding farm	1000	1	62/267
	Feb.	No.13: Pig breeding farm	1100	1	254/682
	Apr.	No.14: Wean to finish	7000	1	93/215
	Apr.	No.15: Self-propagation pig farm with 800 sows	5000	1	103/194
